# NATIONAL INSTITUTE OF TRAUMATOLOGY AND ORTHOPEDICS: A BIBLIOMETRIC STUDY

**DOI:** 10.1590/1413-785220263405e299789

**Published:** 2026-08-10

**Authors:** Rebecca Fiorani de Oliveira Nascimento, João Antonio Matheus Guimarães, Patrícia Cristina dos Santos Costa

**Affiliations:** 1 Divisão de Ensino e Pesquisa (DIENP), Instituto Nacional de Traumatologia e Ortopedia (INTO), Rio de Janeiro, RJ, Brazil.

**Keywords:** Bibliometrics, Scientific Publication Indicators, Orthopedics, Unified Health System, Bibliometria, Indicadores de Produção Científica, Ortopedia, Sistema Único de Saúde

## Abstract

**Objective:**

This study aimed to map the scientific production of the National Institute of Traumatology and Orthopedics (INTO) over its 50 years of history.

**Methods:**

We conducted a descriptive bibliometric study in the PubMed, Scopus, and BVS databases, retrieving 522 articles published between 2007 and 2024. We identified the co-citation networks of INTO-affiliated authors, the keyword networks with the main topics addressed in the publications, and the institution's interaction network with other research and teaching entities.

**Results:**

There was a progressive growth in publications linked to INTO. In the last six years (2019–2024), the annual average number of publications increased by 44.83% compared with the previous period. The keywords with the highest co-occurrence were related to applied research, mainly with an epidemiological profile, but also to different study designs in basic and translational research.

**Conclusions:**

The analysis revealed a higher frequency of collaboration with public universities (UFRJ, UFF, UERJ, and USP). The institution's scientific production presented a well-articulated scientific collaboration network. Part of INTO's mission was evidenced through its work in public health research for health promotion in orthopedics and its contribution to the production of data for the formulation of public policies. ***Level of Evidence V; Descriptive Studies with Qualitative Approach.***

## RESUMO

**Objetivo:**

O objetivo deste artigo foi mapear a produção científica do Instituto Nacional de Traumatologia e Ortopedia (INTO) nos seus 50 anos de história.

**Métodos:**

Realizamos estudo bibliométrico descritivo nas bases de dados Pubmed, Scopus e BVS, sendo obtidos 522 artigos, de 2007 a 2024. Identificamos as redes de co-citação dos autores filiados ao INTO, de palavras-chave, com os principais temas abordados nas publicações, e de interação da instituição com outras entidades de pesquisa e ensino.

**Resultados:**

Houve um crescimento progressivo nas publicações ligadas ao INTO. Nos últimos seis anos (2019-2024), a média anual de publicações aumentou 44,83% em relação ao período anterior. As palavras-chave de maior coocorrência estavam relacionadas à pesquisa aplicada, principalmente de perfil epidemiológico, mas também a diferentes desenhos de estudos em pesquisa básica e translacional.

**Conclusões:**

A análise revelou maior frequência de colaboração com as universidades públicas (UFRJ, UFF, UERJ e USP). A produção científica da instituição apresentou uma rede de colaboração científica articulada. Evidenciou-se parte da missão do INTO com a atuação em pesquisa em saúde pública para a promoção de saúde em ortopedia e sua contribuição na produção de dados para elaboração de políticas públicas. ***Nível de Evidência V; Estudos Descritivos com Abordagem Qualitativa.***

## INTRODUCTION

The Instituto Nacional de Traumatologia e Ortopedia (INTO), affiliated with Brazil's Ministry of Health, is a national reference for the high-complexity treatment of the musculoskeletal system within the Unified Health System (SUS). It also stands out in research and innovation in orthopedics and traumatology, contributing to the formulation of guidelines, clinical protocols, and new surgical techniques in Brazil.

Created in 1973 as the Hospital de Traumato-Ortopedia, following the acquisition of the Hospital Central de Acidentados by the National Institute of Social Security, the institution's trajectory has been marked by pioneering work in several areas of orthopedics. Notably, it performed the first hip arthroplasty with a ceramic prosthesis in 1975. In 1994, it was recognized as a National Institute affiliated with the Health Care Secretariat of the Ministry of Health. Currently, INTO is the only Brazilian hospital to be part of the International Society of Orthopaedic Centers (ISOC), a society that brings together the 19 leading orthopedic hospitals in the world and disseminates best practices and innovations in orthopedic patient care.

Musculoskeletal diseases (MSDs) represent a growing challenge for health systems, affecting the population's quality of life and increasing the demand for specialized care. According to global estimates, in 2017 and 2019 MSDs were among the leading diseases affecting the health of the world's population, being the leading cause of disability^1^. Individuals with MSD are twice as likely to develop other chronic systemic diseases compared with those without these conditions^2^. Although prevalent across all age groups, these conditions represent a substantial demand for rehabilitation services, including in children. In this context, specialized hospitals such as INTO are decisive for structuring the care network and optimizing the resources of the SUS^3^, ensuring greater functional, economic, and social independence for the population throughout life.

Considering INTO's institutional relevance and its scientific production in consolidating health policies and practices, the objective of this study is to map the institution's scientific production over its 50 years of history. Specifically, it will quantify works whose affiliation is attributed to INTO, identify the main topics addressed in the publications, and analyze the institution's interaction network with other research and teaching entities.

## MATERIALS AND METHODS

In September 2024, we used the PubMed, Scopus, and BVS databases to identify articles, with the following inclusion criteria: original scientific articles, reviews, case reports, observational or clinical studies, with no time restriction on the searches. The keywords used were "Instituto Nacional de Traumatologia" and "INTO", combined with the Boolean operators AND and OR. Additionally, in the PubMed database, a specific search was performed using the selection of the institution in the "Affiliation" field. The search strategy used, which retrieved 307 publications, is shown below.

1. (((("Instituto Nacional de Traumatologia e Ortopedia"[Affiliation]) OR ("INTO"[Affiliation])) OR ("National Institute of Traumatology and Orthopedics" AND Brazil)) OR (("National Institute of Traumatology and Orthopaedics"[Affiliation]) AND ("INTO"[Affiliation]))) OR ("Instituto Nacional de Traumatologia e Ortopedia Jamil Haddad (INTO)"[Affiliation])

The same strategy was applied to the BVS and Scopus databases, retrieving 489 and 424 publications, respectively.

The Rayyan software was used to remove duplicates and select the articles according to the criteria. Thus, 1,220 publications were identified. After analysis, 617 publications were confirmed as duplicates and discarded.

**Figure 1:**
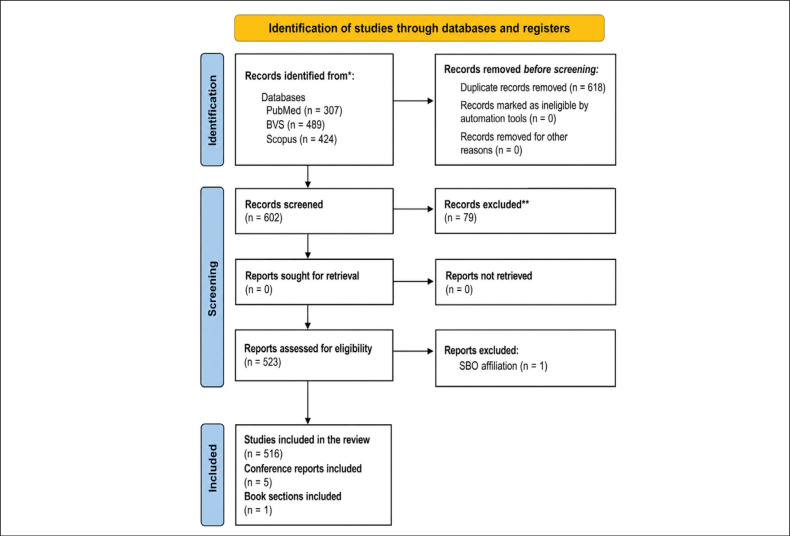
Publication selection flowchart.

Source: Page MJ, et al. BMJ 2021;372:n71. doi: 10.1136/bmj.n71. This work is licensed under CC BY 4.0. To view a copy of this license, visit https://creativecommons.org/licenses/by/4.0/.

Next, the adherence of each publication's affiliation to INTO was verified in 603 publications by two independent reviewers, and disagreements were resolved by discussion. Of these, 79 articles were excluded for being affiliated with the "Instituto Nacional de Traumatologia e Ortopedia" of other countries; one article was excluded because it was conducted at the "Hosp. de Tráumato-Ortop.- RJ (INTO)" but its affiliation was attributed to the Brazilian Society of Orthopedics; and another article was excluded for being a duplicate. Finally, we obtained 522 articles attributed to INTO that were published between 2007 and 2024 (Figure 1).

The bibliometric analysis examined collaboration networks and keyword occurrences with the aid of the VOSviewer software. The evaluation of keywords, authorship, and organizations was preceded by data processing with the Thesaurus tool in order to normalize terms from different languages into English.

## RESULTS

**Figure 2:**
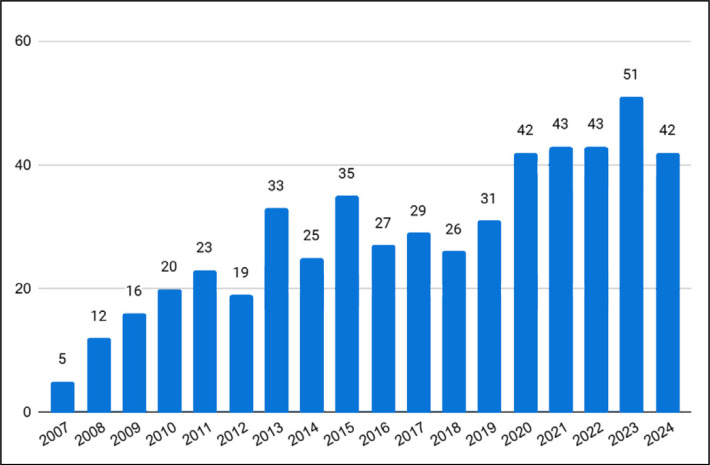
Absolute frequency of the evolution of the scientific production of articles affiliated with INTO from 2007 to 2024.

The annual publication of the 522 articles shows three periods of growth: an initial range up to 2012, followed by a period up to 2018, and finally a new plateau up to 2024 (Figure 2).

The average frequency of scientific publications between 2007 and 2012 was 16 articles per year, increasing to 29 per year from 2013 to 2018. In the last six years (2019–2024), the average rose to 42, a growth of 44.83% compared with the previous period and of 162.5% compared with the first interval analyzed (2007–2012). This significant growth in scientific production reflects a strengthening of institutional research.

**Figure 3:**
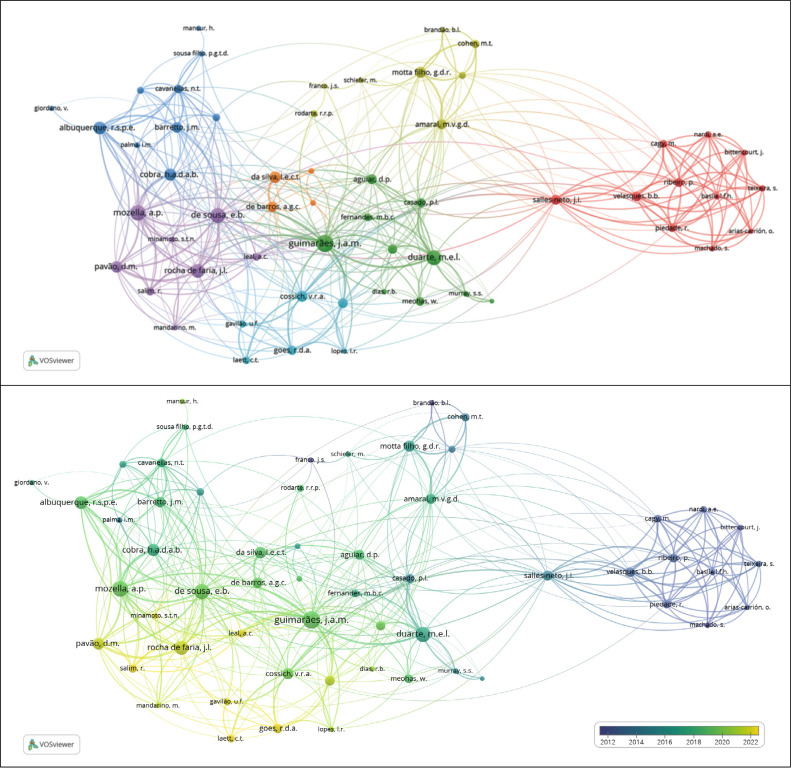
Co-authorship network of authors affiliated with INTO. A: Co-authorship network; B: Temporal co-authorship network.

Regarding author productivity, the analysis of the co-author network can reveal which authors contributed most to research at INTO. Among the 8,982 co-authors who contributed to this research, 58 authors were co-cited more than 95 times. All authors showed a positive co-citation relationship with the other authors. Among the 5 main co-cited authors, the author Guimarães stands out with 980 documents, collaborating with authors present in all 7 clusters generated by VOSviewer (Figure 3A).

*The size of the nodes represents the number of publications of each author in the network, while the thickness of the connections indicates the frequency of collaboration between researchers. More connected authors form clusters, evidencing research groups with greater scientific interaction.

It can be observed in Figure 3A that the 5 main authors belong to two distinct clusters, namely: cluster 2 (Guimarães and Duarte) and cluster 5 (Mozella, De Souza, and Rocha de Faria), indicating groups of authors who frequently collaborate with one another (co-authorship) with high interrelation. Among them, only two establish connections with all 7 clusters identified in VOSviewer, including the author with the highest number of publications and De Souza (fourth place). This indicates that they play a central role in the collaboration network, functioning as links between different research groups, which may reflect interdisciplinary activity or a strong impact within INTO's scientific community. It is interesting to note, when visualizing the publication timeline (Figure 3B), that 4 of the main authors had publications in the last 5 years. Analyzing cluster 1 of the co-authorship network visualization, its separation from the others is evident, indicating that the authors of this group have a more internal collaboration network that is less connected to the other clusters. However, the author Salles-Neto plays a bridging role between this cluster and the others. His proximity to the other nodes suggests that he maintains collaborations both within his own group and with other researchers. This strategic position of Salles-Neto ensures that cluster 1 is not isolated from the other groups.

The co-occurrence analysis of the keywords in the articles published by INTO between 2007 and 2024 identified the main prominent themes within the institution's scientific production. In total, 4,200 keywords were found in 522 articles, with 49 words cited more than 70 times. The VOSviewer analysis allowed these words to be organized into a visual map, in which the proximity between terms indicates their correlation within the field of study. Terms frequently used together form clusters, highlighting specific subareas of research and their thematic connections.

**Figure 4:**
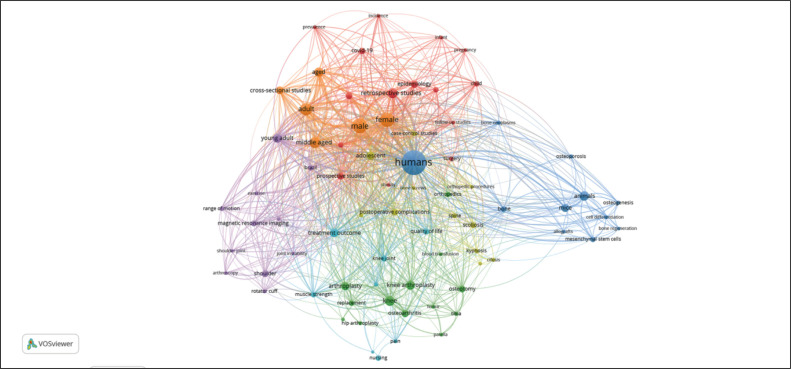
Keyword co-occurrence network.

Figure 4 presents the keywords with the highest co-occurrence. Of these, the five most frequent are related to applied research in humans ("humans", "male", "female", "adult", "middle aged"), reflecting the diversity of the population served at the institution, from children to adults.

*The smaller and more dispersed circles (for example, light blue, purple, and red) represent terms cited with low occurrence frequency and in isolation, such as nursing, arthroscopy, and infant, among others.

A deeper analysis reveals the predominance of terms associated with different observational study designs, highlighting the term "retrospective studies" but also "cross-sectional studies", "prospective studies", "case-control studies", and "cohort studies", suggesting a broad methodological diversity, encompassing cross-sectional and cohort studies with both retrospective and prospective temporal follow-up analyses.

In addition, the formation of a specific cluster related to research with animal models stands out (cluster 4). Figure 4 shows the proximity between the terms "animals" and "mice" and the key terms "mesenchymal stem cells", "osteogenesis", and "osteoporosis", indicating the relevance of developing basic research studies for the implementation of translational research at the institution, and clinical applicability in humans. Interestingly, the terms "animals" and "mice" are connected by the term "bone" as a linking element to the other terms.

In the context of orthopedics, the keywords reflect the activity of the institution's CAAEs, encompassing areas such as Knee ("knee", "knee arthroplasty", "osteotomy", tibia), Hip ("hip arthroplasty", "osteotomy"), Spine ("spine", "scoliosis", "kyphosis"), and Shoulder and Elbow ("shoulder"), evidencing the breadth and high complexity of the research developed at the institution.

The analysis highlights the relative disconnection of cluster 6, formed by the terms "nursing", "pain", and "quality of life". The keyword "nursing" appears isolated, without direct connections to others; its link is made through the term "pain", which connects to 12 keywords.

Finally, a set of words related to the assessment of risk factors ("risk factors"), prevalence ("epidemiology"), outcomes and complications ("treatment outcome", "postoperative complications"), as well as prosthesis and implant interventions ("arthroplasty"), is observed. In addition, terms associated with diagnostic and prognostic assessment examinations ("magnetic resonance image", "radiography", "muscle strength") also stand out, reinforcing the interdisciplinarity of the research developed at the institution, given its eight Research Laboratories registered, such as those for Neuromuscular Research and Exercise Physiology.

**Figure 5:**
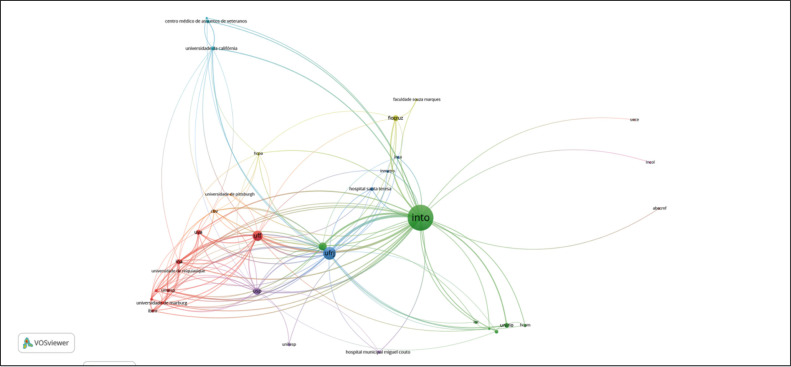
INTO's institutional collaboration network.

Additionally, 34 institutions cited at least 5 times were identified, represented by 305 of the 522 documents retrieved from the databases. Thus, the VOSviewer tool organized these 34 institutions into 10 clusters. Here too, the network map shows (Figure 5) that, in terms of research relationships, despite being within cluster 2, there is strong collaboration between INTO and cluster 1, composed of the National Cancer Institute (INCA), the National Institute of Metrology, Quality and Technology (Inmetro), Hospital Santa Teresa, and the Federal University of Rio de Janeiro (UFRJ). The strong relationship between INTO and cluster 1 is based on the short distance between their nodes^4^.

*The smaller and more dispersed circles (for example, light blue, pink, and purple) represent terms cited with low occurrence frequency and in isolation, such as the Centro Médico de Assunto de Veteranos, Instituto da Coluna Vertebral do Rio de Janeiro (INCOL), Hospital Municipal Miguel Couto, among others.

There is a certain frequency, represented by the lines, with which INTO relates and collaborates in the production of knowledge, with those among the Federal Universities standing out, namely, the Universidade Federal do Rio de Janeiro (UFRJ), the Universidade Federal Fluminense (UFF), and the Universidade Federal do Estado do Rio de Janeiro (UNIRIO); Federal Institutions, such as the Instituto Nacional de Câncer (INCA), the Fundação Oswaldo Cruz (Fiocruz), the Instituto Nacional de Metrologia, Qualidade e Tecnologia (Inmetro), and the Instituto Nacional de Cardiologia (INC); State Universities, such as the Universidade do Estado do Rio de Janeiro (UERJ) and the Universidade de São Paulo (USP); and, finally, among International Institutions and Universities, such as the University of California, the University of Marburg, and the Medical Center for Veterans Affairs, evidencing international insertion through this interaction network. They point to a higher frequency of collaboration with public universities (UFRJ, UFF, UERJ, and USP), especially UFRJ, indicated by the size of the nodes, the short distance between them, and the large thickness of its line (Figure 5).

It can be observed that the relationships between authors for the production of knowledge occurred predominantly between INTO and UFRJ, demonstrating the strong and frequent collaboration between them. Furthermore, it denotes research partnerships among peers in the context of internationalization (University of California, Institute of Applied Neurosciences, and Medical Center for Veterans Affairs) (Figure 5).

## DISCUSSION

Bibliometric studies, in addition to being descriptive tools of scientific production, support internationalization policies by identifying co-authorships with foreign institutions, participation in global networks, and academic excellence criteria^5^. By revealing productivity patterns, collaboration clusters, and knowledge gaps, they guide research-incentive policies, investment planning, and efficient resource allocation. They also make it possible to assess the evolution of performance indicators and guide strategic changes in health services.

In mapping INTO's scientific production, we identified a consistent growth pattern associated with the creation, in 2013, of the Stricto Sensu Graduate Program in Applied Sciences of the Musculoskeletal System. A similar trend had already been observed in the Science, Technology and Innovation Indicators of São Paulo^6^, relating the increase in Brazilian scientific production to the growth of graduate programs, the number of graduate students and degree holders, and the improved qualification of university faculty.

INTO's scientific production has a co-authorship network with a distinct characteristic compared with others described in the literature. While Lima^7^ points out that co-authorship networks tend to be fragmented, composed of several small disconnected components, INTO's network stands out for its broad connectivity, indicating a well-established scientific collaboration structure. This organization suggests the existence of common goals among the researchers, aligned with the orthopedic subspecialties, favoring the exchange of knowledge and the development of more robust research. Moreover, this structuring of collaboration tends to maximize both productivity and scientific impact^8^, and, when combined, they enhance knowledge creation.

It is also possible to observe, in these networks, the correlation between the clusters of researchers and INTO's orthopedic subspecialties. The main authors are currently affiliated with two units at the institution: Guimarães, Duarte, and Rocha de Faria with the Research Division, and Mozella, De Souza, and Rocha de Faria are associated with the Knee Surgery Center. The academic background of these researchers, with experience at UNIRIO, the University of California, UFF, UFRJ, and USP, demonstrates how institutional networks contribute to building collaborative networks, connecting INTO to national and international research centers. In addition, the existence of Graduate Programs, such as the Specialization Course in Nursing, in a Residency format developed in partnership with UNIRIO, may influence interinstitutional collaboration and consequent scientific production.

Although geographic proximity between institutions facilitates the exchange of knowledge and the sharing of resources, scientific collaboration also depends on political and institutional factors, such as regulations, agreements, and cooperation. At INTO, the interaction between specialized groups strengthens scientific production in orthopedics and traumatology, allowing the rapid incorporation of advances across subareas.

Regarding the predominant themes, the keyword with the most appearances is related to applied research in humans. This finding is expected, considering that the institution is a national reference unit of the SUS and is part of the direct administration of the Ministry of Health. This activity is aligned with its vision of being recognized nationally and internationally as a center of excellence in care, teaching, research, and management in the areas of orthopedics, traumatology, and rehabilitation, where scientific findings are directly transposed into clinical practice and into the formulation of health policies.

Furthermore, the results reveal a broad methodological spectrum, with emphasis on translational studies in the health field aimed at overcoming the limitations of conventional bone and cartilage regeneration therapies. Despite advances in the use of biomaterials and mesenchymal stem cells^9^, the clinical application of these approaches still faces challenges related to the standardization of protocols for cell expansion, the low rate of graft integration, and the heterogeneity of the mesenchymal stem cells used^9^. From a public health perspective, understanding how new therapies are integrated into the health system and what barriers exist to their adoption is essential for building evidence-based guidelines.

Another aspect that deserves consideration in the incorporation of new orthopedic therapeutic approaches is the epidemiological profile of the Brazilian population. Between the years 2000 and 2023, the country underwent an accelerated process of population aging; the proportion of older adults jumped from 8.7% to 15.6%, and projections indicate that about 37.8% of Brazilians will be older adults by 2070^10^. With aging, the incidence and prevalence of degenerative processes of the musculoskeletal system11 also increase^11^, affecting the quality of life and functional mobility of this segment of the population.

In the global scenario, data indicate that 40% of older patients undergo orthopedic surgery and, among them, 24% present with sarcopenia, impacting recovery and rehabilitation^12^. In Brazil, hospitalizations due to falls among older adults have been increasing, with a projected economic impact of approximately R$260 million on the Brazilian health system in 2025^13^, reinforcing the need for multidisciplinary and technological approaches to reduce morbidity and improve outcomes^14^.

The distribution of these surgeries across Brazilian territory evidences the concentration of specialized care in a few reference centers, such as INTO. The institution accounts for 58.7% of high-complexity orthopedic surgeries in the city of Rio de Janeiro and 39.3% in the state^15^. Regarding the institution's most common surgeries, they are concentrated in the Spine, Knee, and Hip subspecialties, corroborating the global scenario in which hip and knee arthroplasties are among the most frequently performed elective orthopedic procedures^16^, mainly due to the high prevalence of osteoarthritis and population aging. This care pattern points to the impact of degenerative musculoskeletal diseases and the need for reference centers in the management of these complex conditions.

However, although the SUS provides high-complexity services, demand exceeds its response capacity, resulting in delays in care and worsening of clinical conditions. The importance of strengthening primary health care, capable of acting preventively and diagnostically, therefore stands out^3^.

Another relevant topic for the management of orthopedic patients concerns interdisciplinary discussions. When analyzing the keyword network, a disconnection is observed, especially with the isolation of the term "nursing", revealing a gap regarding the themes of pain and quality of life. In the literature, the role of nursing in pain management and the promotion of self-care is central, especially through educational interventions. For example, preoperative education about pain reduces postoperative opioid consumption and improves patient satisfaction^17^. Continuity of care, especially in chronic conditions^18,19^, and multidisciplinary integration are associated with reduced mortality and long-term sequelae^20^.

These aspects are aligned with the findings of this analysis, which evidenced the strong presence of terms associated with the assessment of risk factors, prevalence, and postoperative complications, in addition to surgical interventions such as arthroplasty.

## CONCLUSION

There was an increase in INTO's scientific production in the period between 2007 and 2024, especially in the last six years (2019–2024). The institution presented a well-articulated scientific collaboration network and highlighted the importance of strategic partnerships in promoting evidence-based practices and in training qualified professionals, reflected in the productivity of its collaborators.

INTO's commitment to public health research, especially in the epidemiological area, and its multidisciplinary approaches were evidenced. In this way, its integration with primary care strengthens the SUS, ensuring more efficient and effective care for the population, which reaffirms INTO's role as a center of excellence in orthopedic care and as an active agent in the promotion of public health in Brazil.

## Data Availability

The data underlying the research text are available upon the reviewers' demand/request.
